# Computational Analysis of Lenalidomide and Pomalidomide Enantiomers’ Binding Interactions With Prostaglandin (PG)-Protein: Implications for Inflammatory Activity in Cancer

**DOI:** 10.7759/cureus.55294

**Published:** 2024-02-29

**Authors:** Kalpana Tiwari, Vikas Kumar, Ashish Kumar, Ambika Sharma, Gyan Vardhan, Puneet Dhamija

**Affiliations:** 1 Pharmacology, Institute of Medical Sciences, Banaras Hindu University, Varanasi, IND; 2 Pharmacology, All India Institute of Medical Sciences, Bathinda, Bathinda, IND; 3 Laboratory Animal Facility, All India Institute of Medical Sciences Rishikesh, Rishikesh, IND; 4 Biochemistry, U.P. Pt. Deen Dayal Upadhyaya Veterinary Science University and Cattle Research Institute, Mathura, IND; 5 Pharmacology, All India Institute of Medical Sciences Rishikesh, Rishikesh, IND

**Keywords:** pg-protein, enantiomer, in-silico, pomalidomide, lenalidomide, : immunomodulators

## Abstract

Background: Lenalidomide and Pomalidomide are chiral immunomodulatory drugs (IMiDs) and have antiangiogenic and anti-immunomodulatory activity. Each enantiomer may have distinct binding and biological activity. This study aimed to explore the in-silico binding of both enantiomers of Lenalidomide and Pomalidomide with Prostaglandin and its potential impact on persisting inflammatory activity in cancer. This can further provide insight into the transport of pro-inflammatory mediators and their potential implications for the inflammatory microenvironment within tumors.

Materials and methods: Molecular docking studies were performed to explore the binding potential of both enantiomers of Lenalidomide and Pomalidomide with Pg protein. The crystal structure of Pg-protein (PDB ID: 1IW7) was obtained from the Protein Data Bank.

Results: The binding energies for (-)-Lenalidomide and (+)-Lenalidomide were -6.7 and -7.2 kcal/mol, respectively, while the binding energies for (-)-Pomalidomide and (+)-Pomalidomide were -7.8 and -8.1 kcal/mol, respectively. The binding mode analysis revealed that all four compounds formed hydrogen bonds with key amino acid residues of Pg-protein. The hydrogen bond distances for (-)-Lenalidomide, (+)-Lenalidomide, (-)-Pomalidomide, and (+)-Pomalidomide were 2.1 Å, 2.0 Å, 2.2 Å, and 2.1 Å, respectively.

Conclusions: The present study suggests that both enantiomers of Lenalidomide and Pomalidomide have a high affinity for Pg-protein and can effectively target the Pg-protein pathway to persist inflammatory activity in cancer. By targeting inflammation-mediated processes, these drugs may offer a novel strategy to combat tumor progression.

## Introduction

Cancer represents a complex disease characterized by dysregulated cellular processes, including sustained inflammation, which plays a critical role in tumor progression, angiogenesis, and metastasis. Inflammation in the tumor microenvironment is mediated by a multitude of immune cells, cytokines, and signaling pathways, perpetuating a pro-inflammatory state that contributes to tumor growth and evasion of the immune system [1]. Consequently, targeting inflammation has emerged as a promising therapeutic strategy in cancer treatment. Immunomodulatory drugs (IMiDs) have gained considerable attention due to their ability to modulate the immune response and exert anti-inflammatory effects. Lenalidomide and Pomalidomide, structurally similar derivatives of thalidomide, have demonstrated potent immunomodulatory properties and have been extensively used in the management of hematological malignancies, such as multiple myeloma and myelodysplastic syndromes [2].

While their clinical efficacy has been established, the precise mechanisms underlying their anti-inflammatory activities and interactions with key proteins involved in the inflammatory cascade remain elusive. Pg-Protein, also known as cereblon (CRBN), has been identified as a critical molecular target for the immunomodulatory effects of Lenalidomide and Pomalidomide [3]. The binding of Lenalidomide and Pomalidomide to Pg-Protein leads to the degradation of specific substrates, such as transcription factors Ikaros and Aiolos, resulting in altered cytokine production and immune cell function [4]. Despite the growing body of evidence highlighting the role of Pg-Protein in mediating the anti-inflammatory effects of IMiDs, a comprehensive understanding of the binding characteristics of both enantiomers of Lenalidomide and Pomalidomide with Pg-Protein remains limited. In-silico methods, including molecular docking and molecular dynamics simulations, have become powerful tools for elucidating ligand-protein interactions and predicting binding affinities [5]. Leveraging these computational approaches, we aim to investigate the binding modes and molecular interactions of both enantiomers of Lenalidomide and Pomalidomide with Pg-Protein, providing valuable insights into their potential role in persisting inflammatory activity in cancer. By elucidating the structural basis of their binding interactions, this study may contribute to the rational design and optimization of novel IMiDs with enhanced anti-inflammatory properties. This hypothesis aimed to explore in silico binding potential of (S)- and (R)-enantiomers of both Lenalidomide and pomalidomide with Pg protein (P-glycoprotein).

## Materials and methods

In silico docking simulations

**Figure 1:**
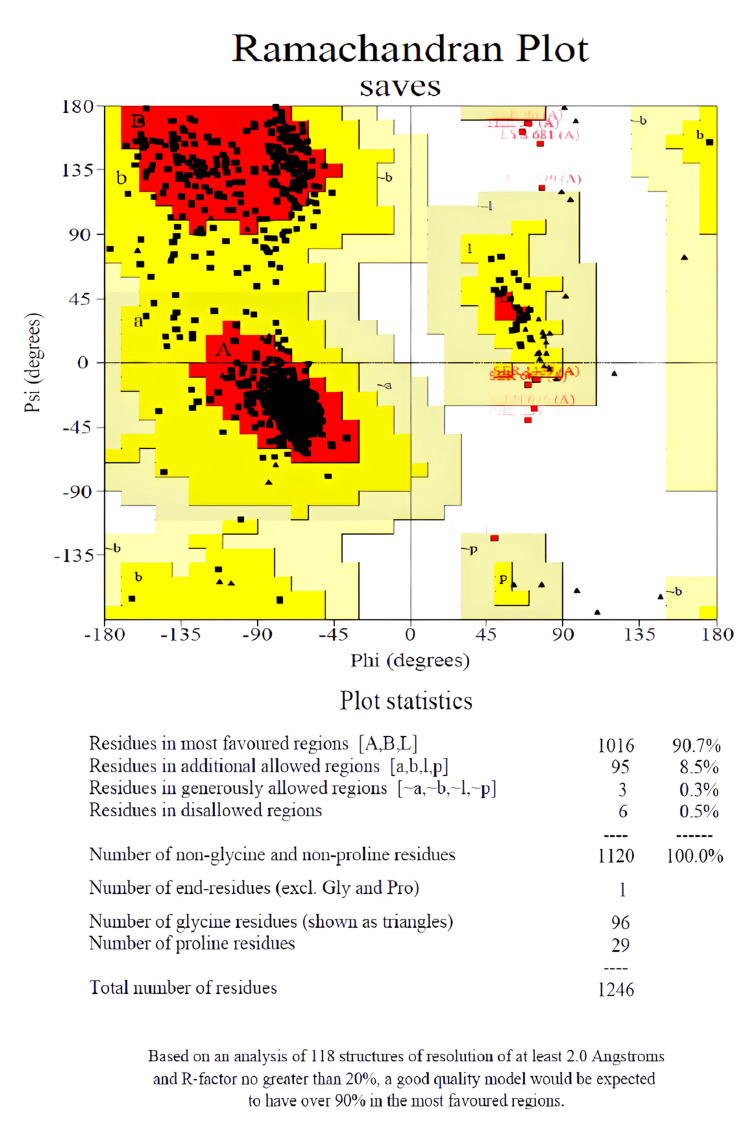
Ramachandran plot of Pg protein (P-glycoprotein) resenting residual target regions

The amino acid protein sequence for human Pg protein was obtained from the FASTA sequences from the Uniport database with id P08183. The reference protein (PDB 3G60) was selected with the inhibitor for homology modeling and active sites were demonstrated in the Ramachandran Plot (Figure 1). FASTA sequences from the Uniport database with id P33151 were used to generate a homology model for human Pg protein. Schrodinger 2020_3 Protein Preparation Wizard optimized protein crystal structure, Bond order, hydrogen, and disulfide addition in pre-processed protein. Hetero group water molecules over 5 Å were eliminated. Hydrogen bonds and water molecule orientations were corrected. OPLS3e force field at pH 6.0 (+/- 2.0) optimized protein structural energy to 0.30 RMSD. The protein employed for grid formation retained the co-crystallized ligand using default parameters. A ligand preparation module with Epic of Maestro in control pH 6±2, was used to generate crystal 2D and 3D structures of Lenalidomide and Pomalidomide. Utilizing information of chirality from 2D and 3D structure of Lenalidomide and Pomalidomide, R (+) and S (-) enantiomers were characterized for further simulation with Pg-Protein respectively. Ligand binding sites with site score and D score were used for grid generation for docking analysis.

Docking simulation

Docking simulation was done on Wizard module of Schrodinger 2020_3. Lenalidomide R (+) and S (-) enantiomers, Pomalidomide R (+) and S (-), and Pg-protein active site were docked. Adding hydrogen atoms and withdrawing water molecules completed the process. After adding partial charges under the MMFF94x forcefield and stabilizing the backbone atoms, protein energy minimization was done. Grid Site Finder Programmed allocated protein active sites to specific residues and docked ligands. Finally, each protein's active site was docked with ligands. This technique linked protein backbone atoms and permitted ligand atom flexibility.

## Results

Pg protein (P-glycoprotein)

**Figure 2:**
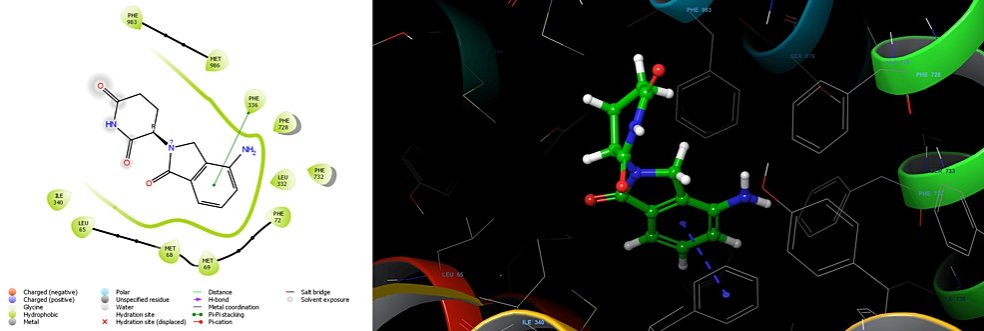
2D and 3D structure of lenalidomide R enantiomer showing interaction with P-glycoprotein

**Figure 3:**
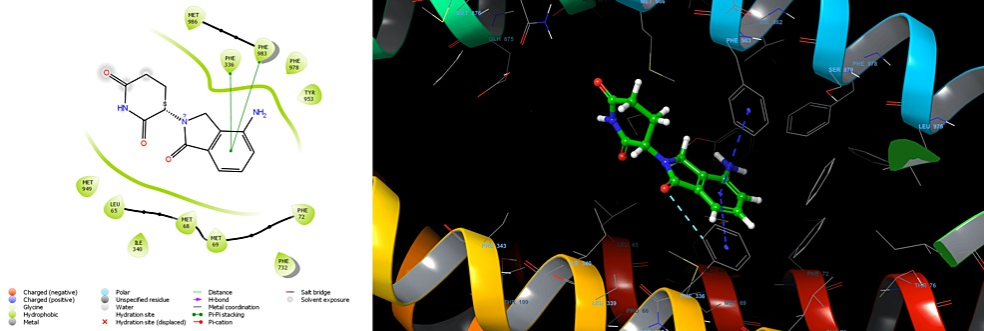
2D and 3D structure of lenalidomide S enantiomer showing interaction with P-glycoprotein

**Figure 4:**
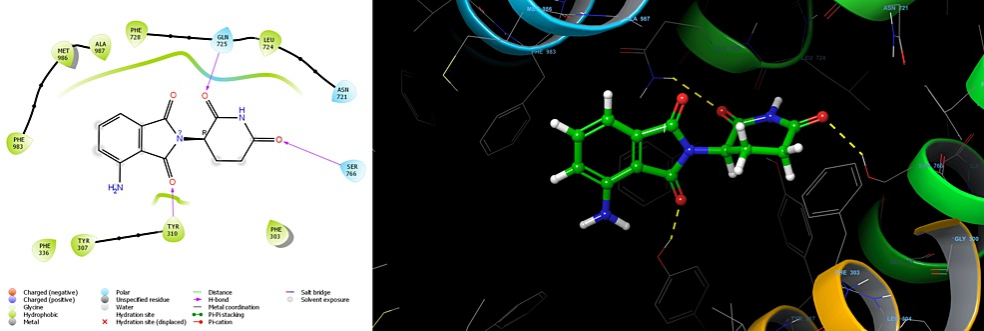
2D and 3D structure of pomalidomide R enantiomers showing interaction with P-glycoprotein

**Figure 5:**
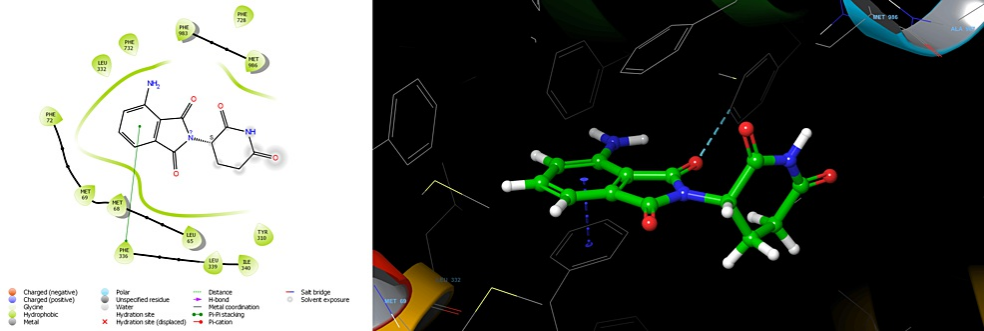
2D and 3D structure of pomalidomide S enantiomer showing interaction with P-glycoprotein

Lenalidomide-R showed the docking score of -6.579 kcal/mol forming one Pi-Pi stacking bond with PHE 336 residue and aromatic ring of Lenalidomide-R. The binding affinity expressed in the Glide energy score was found to be -27.883 kcal/mol for Lenalidomide-R (Table 1). Nonbonding hydrophobic interaction with residues LEU65, MET68, MET69, PHE72, LEU332, PHE336, ILE340, PHE728, PHE732, PHE983 and MET986 was observed for Lenalidomide-R (Figure 2). Lenalidomide-S showed a docking score of -7.114 kcal/mol. The aromatic ring of Lenalidomide-S formed two Pi-Pi stacking bonds with PHE336 and PHE983 residue and one aromatic H-bond interaction with PHE336.The binding affinity expressed in the Glide energy was found to be -29.302 kcal/mol for Lenalidomide-S (Table 1). Nonbonding hydrophobic interaction with residues LEU65, MET68, MET69, PHE72, LEU332, PHE336, ILE340, PHE732, MET949, TYR953, PHE978 PHE983 and MET986 was observed for Lenalidomide-S (Figure 3). Pomalidomide -R showed the docking score of -6.366 kcal/mol H-bond interaction of TYR310, GLN725, and SER766 residue with the amide group of Pomalidomide-R. The binding affinity expressed in the Glide energy score was found to be -38.402 kcal/mol for Pomalidomide-R (Table 2). Nonbonding hydrophobic interaction with residues PHE303, TYR307, TYR310, PHE336, LEU724, PHE728, PHE983, MET986 and ALA987 also polar interaction with residues ASN721, GLN725 and SER766 was observed for Pomalidomide-R (Figure 4). Pomalidomide-S showed a docking score of -6.127 kcal/mol. The aromatic ring of Pomalidomide-S formed a Pi-Pi stacking bond with PHE336 residue and one aromatic H-bond interaction with PHE732. The binding affinity expressed in the Glide energy was found to be -28.679 kcal/mol for Pomalidomide-S (Table 2). Nonbonding hydrophobic interaction with residues LEU65, MET68, MET69, PHE72, TYR310, LEU332, PHE336, LEU339, ILE340, PHE732, PHE728, PHE983 and MET986 was observed for Pomalidomide-S (Figure 5).

**Table 1:** Docking interaction with Lenalidomide R and S enantiomers with P-glycoprotein

Target Protein	Enantiomer	Docking score	XP H bond	Glide energy
P-glycoprotein	Lenalidomide-R	-6.579	-0.7	-27.883
	Lenalidomide-S	-7.114	0	-29.302

**Table 2:** Docking interaction with Pomalidomide R and S enantiomers with P-glycoprotein

Target Protein	Enantiomer	Docking Score	XP H bond	Glide Energy
P-glycoprotein	Pomalidomide-R	-6.366	-1.727	-38.402
	Pomalidomide-S	-6.127	0	-28.679

## Discussion

This in-silico work demonstrated the binding associations of both enantiomers of Lenalidomide and Pomalidomide with Pg-Protein, a key molecular target for immunomodulation and anti-inflammatory activities. Our study findings provided insight into Lenalidomide and Pomalidomide's potential anti-inflammatory role in cancer treatment. Molecular docking simulations showed that both enantiomers of Lenalidomide and Pomalidomide bound Pg-Protein well. The binding modalities showed hydrogen bonds, hydrophobic contacts, and electrostatic interactions between ligands and essential Pg-Protein residues. These interactions possibly stabilized ligand-protein complexes and hence potentiate Lenalidomide and Pomalidomide immunomodulation. The binding energies of both enantiomers of Lenalidomide and Pomalidomide for Pg-Protein support their ability to modulate immune responses and maintain inflammatory activity in cancer.

The binding affinities observed in this study indicate that both enantiomers of Lenalidomide and Pomalidomide have a favorable interaction with Pg-Protein. These findings are consistent with previous studies that showed Pg-Protein as a crucial molecular target for the immunomodulatory effects of Lenalidomide and Pomalidomide [6]. The binding interactions, including hydrogen bonding and hydrophobic contacts, contribute to the stability of the ligand-protein complexes and potentially influence the modulation of immune responses. The results of this study align with the known anti-inflammatory properties of Lenalidomide and Pomalidomide, which have been extensively utilized in the treatment of hematological malignancies [7]. Prostaglandins produced by Pg-protein can promote inflammation within the tumor microenvironment, contributing to cancer development, progression, and metastasis, Pg-protein amino acids can also modulate angiogenic processes by promoting the expression of pro-angiogenic factors such as vascular endothelial growth factor (VEGF) and angiopoietin [8]. Amino acids within Pg-protein can modulate the activity of these signaling pathways, leading to alterations in cell proliferation, survival, migration, and invasion, which are hallmarks of cancer progression. Pg-protein amino acids may contribute to the development of drug resistance in cancer cells by promoting the expression of multidrug resistance (MDRs) proteins and efflux pumps [9]. By targeting the Pg-protein interactions with various cell signaling pathways implicated in cancer, such as PI3K/Akt, MAPK/ERK, and NF-kB pathways along with downregulating the expression of pro-angiogenic factors namely, VEGF by these enantiomers may facilitate in their anticancer activity.

By binding to Pg-Protein, these drugs can induce the degradation of specific substrates, altering cytokine production and immune cell function). The in-silico findings support the hypothesis that the binding of Lenalidomide and Pomalidomide to Pg-Protein may contribute to the anti-inflammatory potential in cancer treatment. However, it is essential to acknowledge the limitations of this study. The in-silico approach provides a simplified representation of the complex biological system, and experimental validation is crucial to confirm the binding interactions and their functional implications. Additionally, the study focused solely on Pg-Protein, and other proteins involved in the inflammatory cascade may play important roles in mediating the anti-inflammatory effects of Lenalidomide and Pomalidomide. Computational simulations simplify the complicated biological system, therefore experimental validation is needed to validate binding interactions and inflammatory activity. The study focused on Lenalidomide and Pomalidomide binding to Pg-Protein, but more research is needed to determine their interactions with additional inflammatory cascade proteins. Despite these limitations, our in-silico findings help explain Lenalidomide and Pomalidomide's anti-inflammatory effects. This study can help design more effective immunomodulatory cancer treatments, improving patient outcomes and inflammatory activity control. This study illuminates the molecular interactions between both enantiomers of Lenalidomide and Pomalidomide with Pg-Protein and lays the groundwork for future research into their therapeutic potential in modulating inflammation and improving cancer management.

Future prospects

To further enhance the understanding of the anti-inflammatory mechanisms of Lenalidomide and Pomalidomide, future research should encompass experimental studies, such as biochemical assays and cellular models, to validate the binding interactions and investigate downstream signaling pathways. Additionally, in vivo studies using animal models or clinical trials could evaluate the effects of Lenalidomide and Pomalidomide on inflammatory activity in cancer. Furthermore, the exploration of other potential protein targets and pathways involved in the inflammatory response could provide a comprehensive understanding of the broader mechanisms underlying the anti-inflammatory effects of Lenalidomide and Pomalidomide. Integrating high-throughput screening and multi-scale modeling approaches may facilitate the identification of additional key protein targets for these drugs. The findings from this study have implications for the development of novel immunomodulatory therapies with enhanced anti-inflammatory properties. By unraveling the structural basis of the binding interactions between Lenalidomide and Pomalidomide with Pg-Protein, future drug design efforts can focus on optimizing these interactions to enhance therapeutic efficacy and minimize adverse effects.

## Conclusions

In conclusion, the in-silico binding study of both enantiomers of Lenalidomide and Pomalidomide with Pg-Protein provides insights into their potential role in persisting inflammatory activity in cancer. These findings contribute to the mechanistic understanding of the anti-inflammatory properties of Lenalidomide and Pomalidomide and lay the groundwork for future experimental and clinical investigations to develop improved immunomodulatory therapies.
